# Thermotaxis in *Chlamydomonas* is brought about by membrane excitation and controlled by redox conditions

**DOI:** 10.1038/s41598-018-34487-4

**Published:** 2018-10-31

**Authors:** Masaya Sekiguchi, Shigetoshi Kameda, Satoshi Kurosawa, Megumi Yoshida, Kenjiro Yoshimura

**Affiliations:** Department of Machinery and Control Systems, College of Systems Engineering and Science, Shibaura Institute of Technology, Saitama, 337-8570 Japan

## Abstract

Temperature is physiologically critical for all living organisms, which cope with temperature stress using metabolic and behavioral responses. In unicellular and some multicellular organisms, thermotaxis is a behavioral response to avoid stressful thermal environments and promote accumulation in an optimal thermal environment. In this study, we examined whether *Chlamydomonas reinhardtii*, a unicellular green alga, demonstrated thermotaxis. We found that between 10 °C and 30 °C, *Chlamydomonas* cells migrated toward lower temperatures independent of cultivation temperature. Interestingly, when we applied reagents to change intracellular reduction-oxidation (redox) conditions, we saw that thermotaxis was enhanced, suppressed, or reversed, depending on the redox conditions and cultivation temperature. Thermotaxis was almost absent in *ppr2* and *ppr3* mutants, which cannot swim backward because of a defect in generating calcium current in flagella. The frequency of spontaneous backward swimming was lower at more favorable temperature, suggesting a pivotal role of spontaneous backward swimming generated by flagellar membrane excitation.

## Introduction

Temperature is a key environmental factor for living organisms because chemical reaction rates and physical characteristics of biological materials can change substantially with temperature. Living organisms acclimate to cold and heat stress using acquired mechanisms, including the ability to migrate to an environment with temperatures suitable for inhabitation. One of the simplest forms of the behavior to migrate to a suitable thermal environment is thermotaxis. Thermotaxis has been found in multicellular organisms, such as *Caenorhabditis elegans* and *Drosophila melanogaster*, as well as in unicellular organisms, such as *Paramecium caudatum*, *Dictyostelium discoideum*, *Physarum polycephalum*, and *Escherichia coli*^1^. Individual cells within multicellular organisms also show thermotaxis. For example, mammalian sperm migrate through the oviduct to the fertilization site guided by a rise in temperature^2^.

The investigation of how unicellular organisms migrate toward preferred temperatures began more than 100 years ago^1^. In particular, the thermotactic behavior of *Paramecium* cells has been well studied. *Paramecium* cells accumulate at sites that are close to the cultivation temperature, i. e. the temperature at which cells are grown^1^. Accumulation at these sites occurs because cells frequently reverse their swimming direction when they encounter a temperature change that deviates from the cultivation temperature and increase their swimming velocity when they experience a temperature change that approaches the cultivation temperature^3,4^. The reversal in swimming direction is induced by a depolarizing receptor potential, which triggers an action potential in the cilia^5^. These studies on *Paramecium* cells highlighted the thermotaxis in unicellular organisms more than 30 years ago, but the molecular mechanisms for thermoreception and signal transduction are not yet understood.

The understanding of the molecular mechanisms for thermotaxis has progressed greatly in recent years, from investigations of mammalian sperm. Human sperm migrates toward warmer temperatures, ranging from 29 °C to 41 °C^2^. Sperm can detect a temperature gradient as small as 0.014 °C/mm, suggesting that sperm detect temporal changes in temperature rather than spatial differences^2^. Several molecules have been proposed to be sensor molecules, including opsin and transient receptor potential (TRP) channels such as TRPV1, TRPV4, and TRPM8^6–9^. TRP channels are multimodal sensor for thermal, chemical and mechanical stimuli, but the function of opsins as a thermosensor awaits to be established.

Temperature is a critical environmental factor also for *Chlamydomonas* cells, which produce small heat shock proteins, chaperonins, and HSP70 heat shock proteins, and also undergo other heat shock responses to cope with heat stress^10–13^. In response to a cold shock of 4 °C, cells halt proliferation and accumulate starch and sugar^14^. Behavioral responses to avoid stressful warm or cold environments are expected to be present in *Chlamydomonas*. Although *C. moewusii* cells are reported to migrate toward warmer temperatures in a 10 °C–15 °C gradient^15^, there has been no report in which the temperature range was systematically manipulated to examine a relationship with cultivation temperature.

In contrast to thermotaxis, phototaxis has been extensively studied in *Chlamydomonas*. Two flagella of *Chlamydomonas* beat in a breast-stroke like pattern during forward swimming and, during phototaxis, *Chlamydomonas* cells make by a turn toward or away from a light source by controlling the balance of the propulsive forces generated by the two flagella^16,17^. The balance depends on the intraflagellar calcium ion concentration; thus, loss of calcium-dependent control in *ptx1* mutants results in a phototaxis defect^18–20^. The direction of phototaxis in *Chlamydomonas* depends on the light intensity, but is also affected by intracellular reduction-oxidation (redox) conditions^21^. Cells migrate toward a light source when the light intensity is weak, but the direction reverses under reducing conditions. In contrast, cells swim away from light sources with strong intensity, but the direction reverses under oxidizing conditions. However, it is not fully understood how ubiquitous the influence of redox state is on cell behavior. Thus, in this study we investigate whether redox state affects thermotaxis.

Our use of *C. reinhardtii* for this study has the potential to advance the understanding of thermotaxis because a wide range of molecular biology tools, including the whole genome sequence, an indexed and mapped mutant library, and CRISPR/Cas9 gene editing method are available for this organism^22–24^. It is noteworthy that seven opsins and at least eight TRP channels are expressed in *Chlamydomonas* cells, considering the involvement in sperm thermotaxis^25^. Further, a large number of characterized *Chlamydomonas* mutants can support the analysis of the involvement of specific functions and genes in a given process.

In this study, we assessed thermotaxis in *C. reinhardtii* and examined how their movement correlated with the cultivation temperature. In contrast to *Paramecium* and *C. moewusii*, *C. reinhardtii* cells migrated toward lower temperatures between 10 °C and 30 °C, irrespective of the cultivation temperature. We also tested whether the thermotaxis was affected by intracellular redox conditions and found that thermotaxis was greatly affected by redox states. Lastly, we used mutants defective for flagellar motility control to investigate the mechanism for thermotaxis.

## Results

### Negative thermotaxis occurs in various temperature gradients

**Figure 1:**
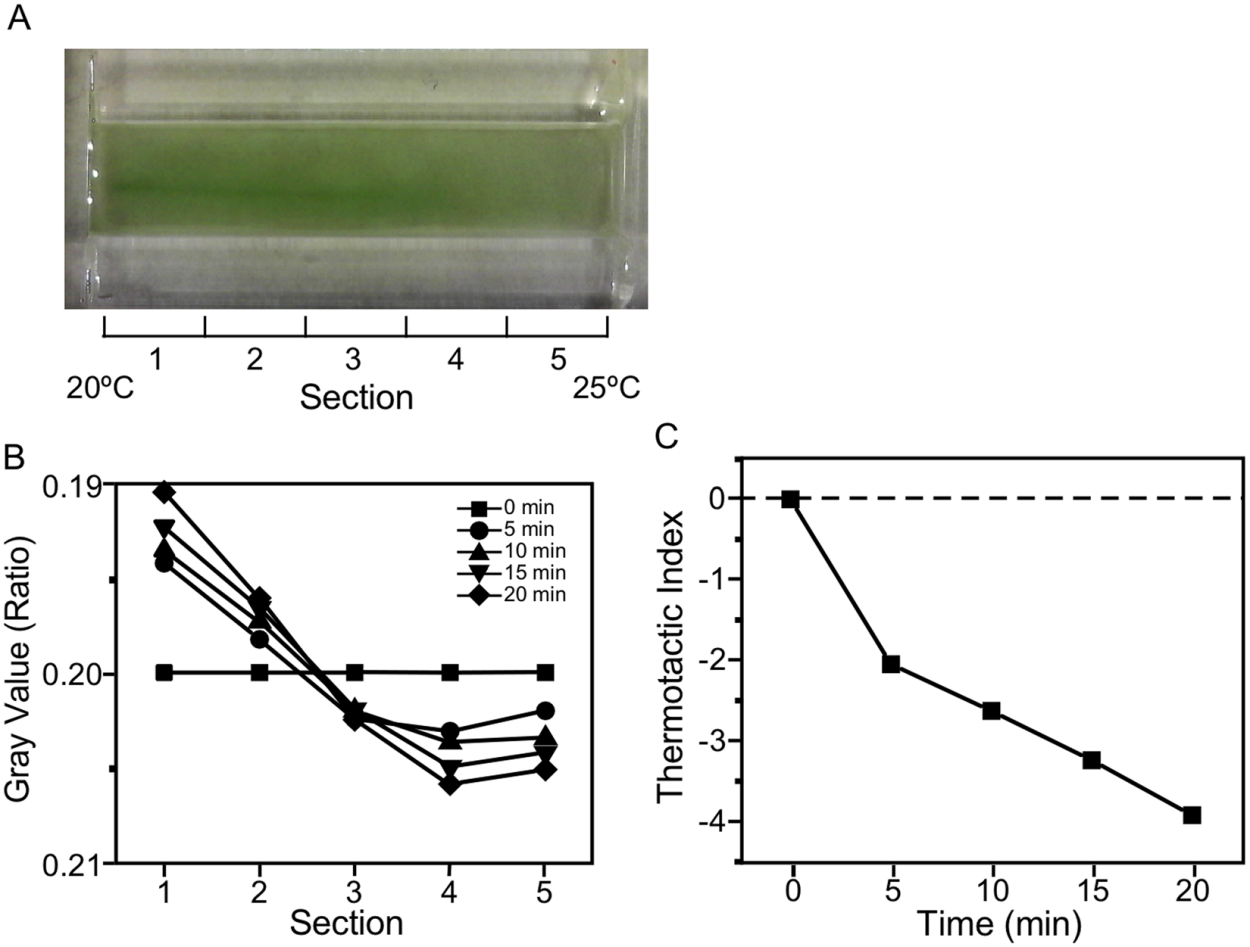
Response to 20 °C–25 °C temperature gradient in the wild type *Chlamydomonas* cells grown at 25 °C. (**A**) Top view of cell suspension in trough taken 20 min after cell suspension was placed under the temperature gradient. (**B**) Cell density along the length of the trough, divided into five sections. Cell density was obtained by gray scale and normalized to the average value at time = 0 min. The density was obtained 0 to 20 min after incubation in the 20 °C–25 °C temperature gradient. Note that larger and smaller gray values (brighter and darker images) indicate less and more cells, respectively, and that the axis is inverted. (**C**) Time course of the changes in thermotactic index, using data shown in (**B**).

*Chlamydomonas* cells are usually cultured at 25 °C in laboratories, because they grow fastest around this temperature. When we generated a 5 °C temperature gradient from 20 °C to 25 °C in a trough and placed the wild type cells in it, we found that cells accumulated toward the 20 °C side (Fig. 1A). We evaluated the cell distribution by converting the cell density to gray scale and obtaining the mean gray scale for five sections. We observed an increase in cell density in two sections with lower temperatures 5 min after the onset of temperature gradient, whereas the cell density decreased in three sections with higher temperatures (Fig. 1B). We saw that this tendency progressed at least for 20 min. The bias of cell distribution was expressed as thermotactic index, in which zero indicates that the distribution is even, and positive or negative values indicate that the distribution is biased toward higher or lower temperatures, respectively. The use of the thermotactic index confirmed that negative thermotaxis (i.e. migration toward lower temperatures) occurred during this period (Fig. 1C).

**Figure 2:**
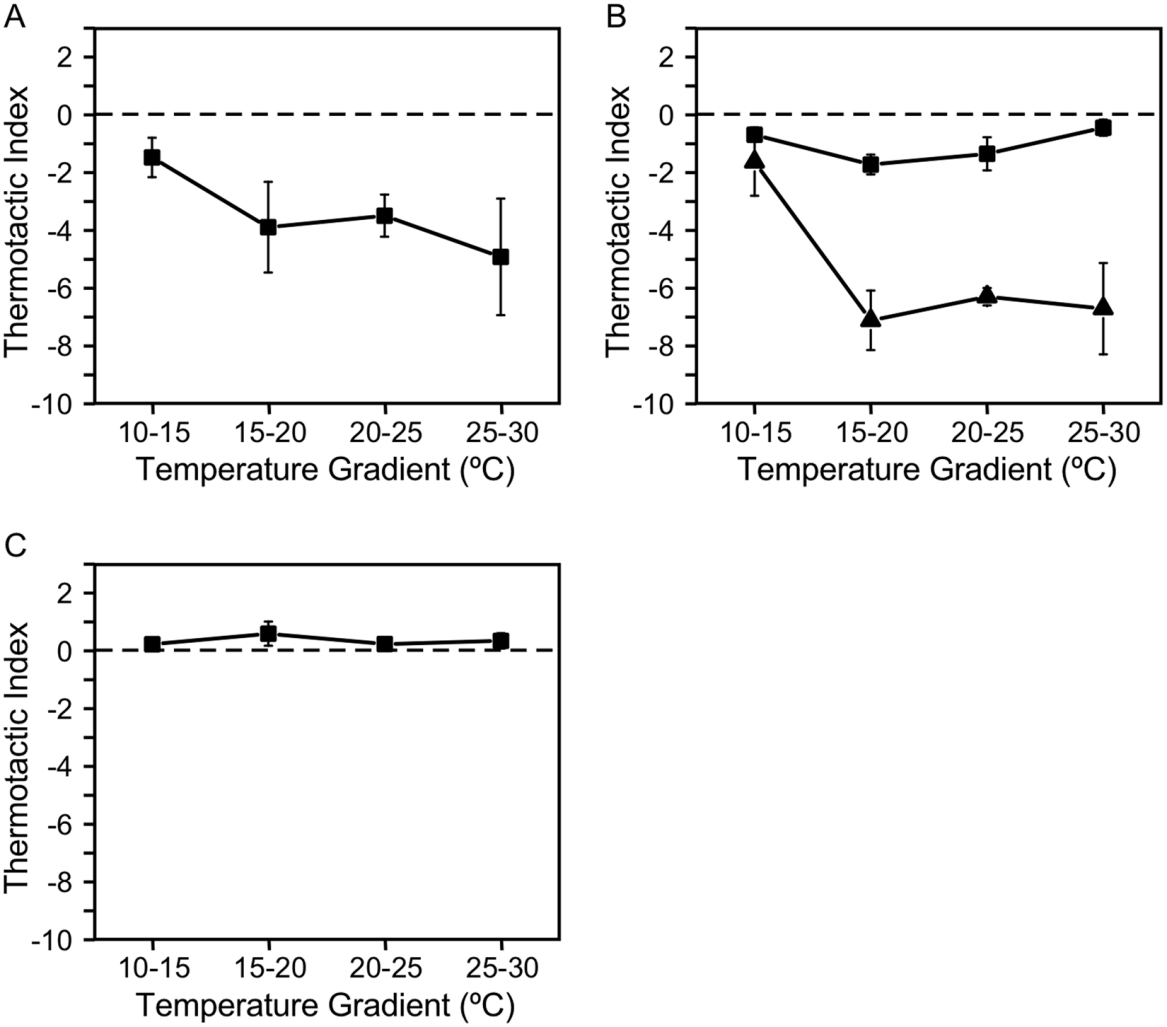
Responses to different temperature gradients in wild type cells grown at (**A**) 25 °C, (**B**) 20 °C (square), and 15 °C (triangle). (**C**) Response of an immotile mutant, *pf17*. Data represent mean ± standard error of the mean (SEM) of three experiments.

When cells cultured at 25 °C were placed in a 5 °C temperature gradient of 10 °C–15 °C, 15 °C–20 °C, 20 °C–25 °C, and 25 °C–30 °C gradients, cells demonstrated negative thermotaxis at all temperature levels (Fig. 2A). The magnitude of negative thermotaxis was comparable between 15 °C–20 °C, 20 °C–25 °C, and 25 °C–30 °C gradients, but was smallest with the 10 °C–15 °C temperature gradient.

Because the direction of thermotaxis in *Paramecium* depends on the cultivation temperature^1^, we further examined thermotaxis in cells cultured at 15 °C and 20 °C. Cells cultured at 15 °C and 20 °C also showed negative thermotaxis at all temperatures (Fig. 2B). However, the magnitude of movement was greater in cells grown at 15 °C and modest in cells grown at 20 °C, compared to thermotaxis in cells grown at 25 °C. Thermotaxis in the 10 °C–15 °C temperature gradient was the weakest, for cells grown at any temperature.

To confirm that the bias in cell density was not caused by a physical artifact, such as convection, we applied the above protocol to an immotile mutant, *pf17*. The cell density remained uniform for 20 min, resulting in thermotactic index of practically zero (Fig. 2C).

### Thermotaxis is affected by redox conditions

**Figure 3:**
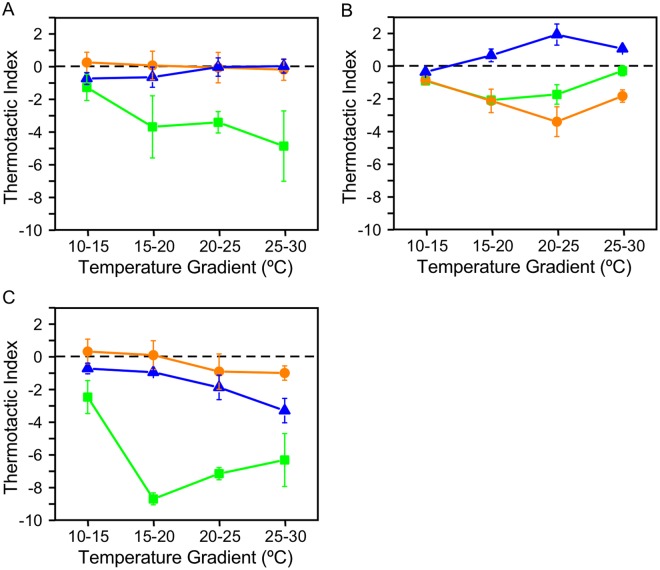
Responses to different temperature gradients with different redox conditions in wild type cells grown at (**A**) 25 °C, (**B**) 20 °C, and (**C**) 15 °C. Thermotactic index of the data obtained under ambient (green), reducing (blue), and oxidizing (orange) conditions is shown. Data represent mean ± SEM of three experiments.

**Figure 4:**
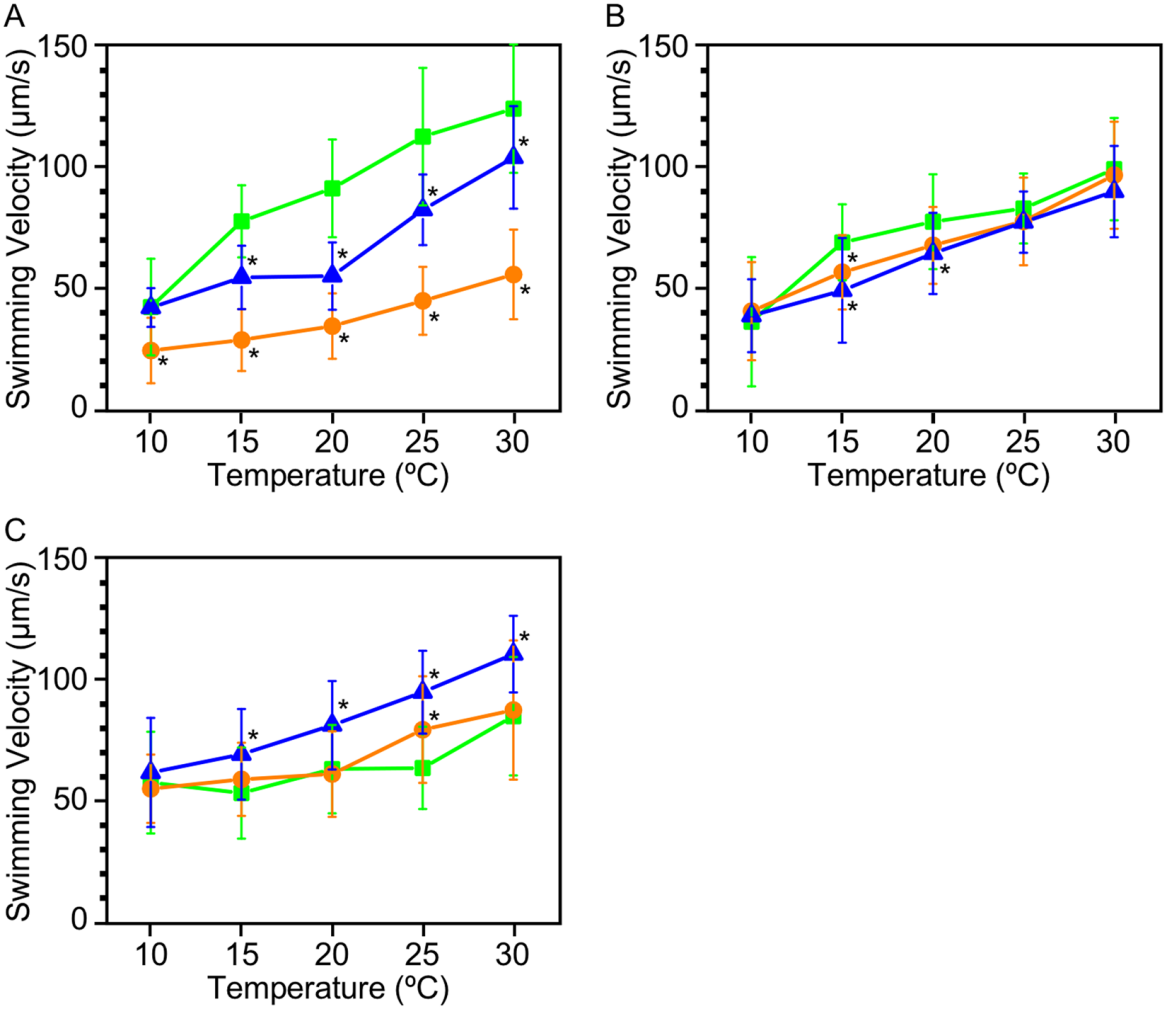
Swimming velocity at different temperatures in wild type cells grown at (**A**) 25 °C, (**B**) 20 °C, and (**C**) 15 °C. Data obtained under ambient (green), reducing, (blue), and oxidizing (orange) conditions are shown. Data represent mean ± SD of 30 cells. Asterisks indicate significant difference (p < 0.01, t-test) when compared with the data obtained in ambient condition.

The direction of phototaxis of *Chlamydomonas* depends on light intensity, but is also affected by intracellular redox conditions^21^. When we subjected cells grown at 25 °C to reducing conditions by addition of 1,3-dimethylthiourea (DMTU), thermotaxis was almost completely lost (Fig. 3A). Thermotaxis also diminished substantially when we subjected cells to oxidizing conditions by addition of *tert*-butyl hydroperoxide (*t*-BOOH). This was not due to a loss of motility, because cells were motile, although with a reduced swimming velocity (see Fig. 4). When cells grown at 20 °C were placed in reducing conditions, we observed positive thermotaxis in 15 °C–20 °C, 20 °C–25 °C, and 25 °C–30 °C gradients (Fig. 3B). Conversely, negative thermotaxis was enhanced in oxidizing conditions. Thus, the direction of thermotaxis reversed, depending on the redox condition, as observed in phototaxis^21^. Additionally, cells grown at 15 °C showed weaker negative thermotaxis in reducing and oxidizing conditions, compared with thermotaxis under ambient conditions (Fig. 3C).

Thus, the result shows a marked impact of redox balance and cultivation temperature on thermotaxis.

### Swimming velocity

The observations above might result from low motility, which could cause cell accumulation^26^. To examine this possibility, we measured the swimming velocity of cells. We found that the swimming velocity of cells grown at 25 °C increased with temperature from 42 µm/s to 123 µm/s, in a range from 10 °C to 30 °C (Fig. 4A). In reducing conditions, the swimming velocity was slightly lower, and in oxidizing conditions, the velocity was less than half that of cells in ambient condition (e.g. 112, 82, and 45 µm/s in ambient, reducing, and oxidizing conditions, respectively, when assessed at 25 °C).

The effect of redox conditions on swimming velocity was almost negligible in cells grown at 20 °C (Fig. 4B) (e.g. 83, 78, and 78 µm/s in ambient, reducing, and oxidizing conditions, respectively, when assessed at 25 °C). Therefore, the reversal or enhancement of thermotaxis in reducing or oxidizing conditions, respectively, is likely not attributable to a dependence of swimming velocity on temperature.

We found that cells grown at 15 °C swam slightly faster in the reducing conditions than in oxidizing or ambient conditions (Fig. 4C) (e.g. 63, 94, and 79 µm/s in ambient, reducing, and oxidizing conditions, respectively, when assessed at 25 °C). Notably, cells in ambient condition showed pronounced thermotaxis in the 15 °C–20 °C gradient (Fig. 3C), but their swimming velocity did not differ between 15 °C and 20 °C. Additionally, the temperature dependence of swimming velocity was very similar between the oxidizing and ambient conditions, despite a great difference in the magnitude of thermotaxis.

Theoretically, the density of moving particles is proportional to the inverse of speed^26^. The correlation of the inverse of swimming speed (1/υ[T]) with temperature (T) supported overall tendency of higher cell density at lower temperatures (Supplementary Fig. 1). The ratio of 1/υ[T] with 5 °C difference, i. e. (1/υ[T])/(1/υ[T + 5]), was calculated to estimate the accumulation toward lower temperatures (Supplementary Fig. 2). The ratio was almost exclusively larger than one, indicating accumulation toward lower temperature. However, the graph did not reproduce the remarkable thermotaxis in ambient condition in cells grown at 25 °C and 15 °C or positive thermotaxis in cells grown at 20 °C, confirming that thermotaxis cannot be explained fully by the temperature-dependent change in swimming velocity.

### Thermotaxis is not observed in mutants with defects in flagellar motility control

**Figure 5:**
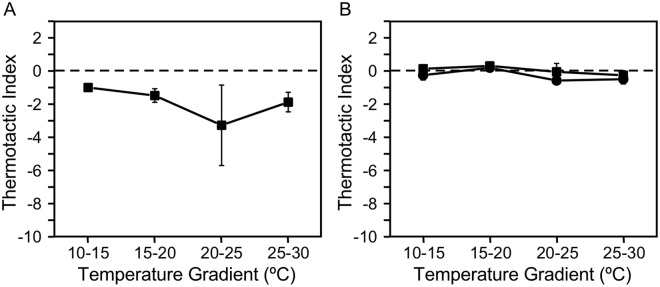
Response to temperature gradients in (**A**) a phototaxis mutant, *ptx1*, and (**B**) photophobic-response mutants, *ppr2* (squares), and *ppr3* (circle). Cells were grown at 25 °C. Data represent mean ± SEM of three experiments.

Phototaxis in *Chlamydomonas* is mediated by calcium-dependent control of flagellar dominance, which steers the cell^18^. Here, we analyzed whether a similar flagellar dominance mechanism is involved in thermotaxis, by using a *ptx1* mutant that is defective for dominance control^19,20^. When we placed *ptx1* cells grown at 25 °C in a 5 °C temperature gradient, we observed thermotaxis in the 20 °C–25 °C gradient (Fig. 5A). We also observed thermotaxis of *ptx1* cells in the 10 °C–15 °C, 15 °C–20 °C, and 25 °C–30 °C gradients, although it was weaker than that of the wild type cells. Therefore, calcium-dependent control of flagellar dominance is probably not essential to thermotaxis.

In *Paramecium*, thermotaxis is mediated by the reversal of swimming direction when cells begin to swim away from their respective cultivation temperature. To examine the involvement of swimming reversal in *Chlamydomonas* thermotaxis, we used *ppr2* and *ppr3* mutants, which are defective for reversing swimming direction^27,28^. *Chlamydomonas* cells generate an all-or-none type flagellar current upon intense light or mechanical stimulation and convert flagellar bending pattern from the breast-stroke-like forward mode to the sinusoidal reverse mode^29^, but these mutants do not generate the flagellar current. When *ppr2* cells were grown at 25 °C and placed in a 5 °C temperature gradient, we did not observe thermotaxis in any gradient from 10 °C to 30 °C (Fig. 5B). We obtained a similar result with the *ppr3* mutant.

**Figure 6:**
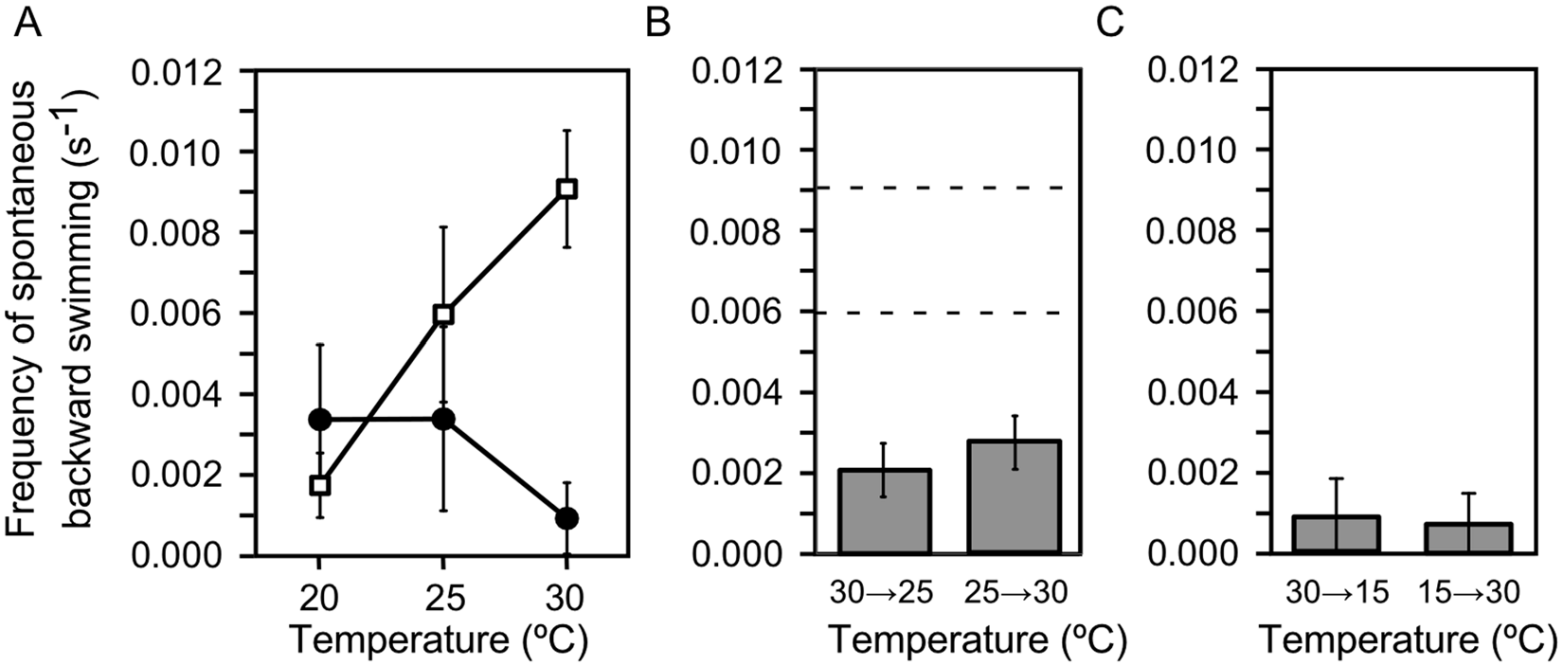
Frequency of spontaneous backward swimming. (**A**) Temperature was kept constant. Cells were grown at 25 °C and examined in ambient condition (open squares) and grown at 20 °C and examined in reducing condition (closed circles). (**B**) Temperature was changed temporally from 30 °C to 25 °C and vice versa at a rate of ±0.01 °C/s. Cells were grown at 25 °C. The upper and lower dashed lines show the frequency obtained at constant temperatures of 30 °C and 25 °C as shown in A. (**C**) Temperature was changed from 30 °C to 15 °C and vice versa at a rate of ±0.14 °C/s. Data represent mean ± SEM of ten experiments in A and six experiments in B and C.

The defect in thermotaxis in *ppr* mutants suggests that backward swimming is involved in thermotaxis. Thus, we tested whether the frequency of spontaneous backward swimming changes with temperature. The distance between the slide and cover glass was kept at ~100 µm and the focus was set in the middle of them to exclude counting avoiding reaction, which occurs on collision with glass surface. We compared the cells grown at 25 °C and kept in ambient redox condition and those grown at 20 °C and kept in reducing condition since the former shows pronounced negative thermotaxis and the latter shows weak positive thermotaxis. The frequency of spontaneous backward swimming increased with temperature in the cells grown at 25 °C and kept in ambient condition but, notably, the frequency did not increase with temperature in cells grown at 20 °C and kept in reducing condition (Fig. 6A).

Since *Paramecium* cells increase the frequency of avoiding reaction when temperature changes away from the cultivation temperature, we observed spontaneous backward swimming during cooling and warming. If we assume that cells swim across a 5 °C temperature gradient at speed of 100 µm/s, they should experience a temporal temperature change rate of approximately 0.016 °C/s. When the temperature was lowered from 30 °C to 25 °C at a rate of 0.01 °C/s, the spontaneous backward swimming occurred at a rate as low as approximately 0.002 s^−1^ (Fig. 6B). When the temperature was increased from 25 °C to 30 °C at the same rate, spontaneous backward swimming occurred at a comparable frequency. These rates were lower than those at stationary temperature of 25 °C and 30 °C (Fig. 6A) suggesting that spontaneous backward swimming was suppressed when cells encountered a temporal temperature change. The suppression was more evident when the temperature was changed at a higher rate (0.14 °C/s, Fig. 6C).

The above observations imply that the frequency of spontaneous backward swimming changes with the temperature in accordance with the direction of thermotaxis at least in part, but does not depend on temporal temperature change. Since backward swimming is induced by a Ca^2+^ influx, it is possible that thermotaxis would not occur in a Ca^2+^-free medium. Such experiment was not presented here because the motility of *Chlamydomonas* cells was impaired in a Ca^2+^-free medium.

## Discussion

Our experiments showed that *Chlamydomonas* cells migrated toward lower temperature in a range between 10 °C and 30 °C. We also found that the redox state greatly affected thermotaxis. Notably, mutants defective in displaying backward swimming were deficient in thermotaxis.

It is unlikely that the biased cell distribution was due to a physical artifact, such as convection, based on following reasons. First, we observed that immotile mutants maintained a uniform distribution under the tested temperature gradients. Secondly, we observed different degrees and directions of bias under the same temperature gradient. For example, cells cultured at 15 °C and 25 °C showed a large distribution bias under ambient conditions, but showed minimal bias under reducing and oxidizing conditions. Cells cultured at 20 °C migrated toward lower temperatures under oxidizing conditions, but toward higher temperatures under reducing conditions. These observations indicate that the bias is a physiological response rather than a physical artifact. It is also unlikely that the absence of thermotaxis in *ppr2* and *ppr3* was due to experimental error, because we observed clear thermotaxis in the positive control experiment on wild type cells, which used the same solution and trough on the same day.

*Chlamydomonas* cells favored lower temperatures in ambient redox conditions for all cultivation temperatures that were tested. Häder *et al*.^30^ summarized that “*Haematococcus* and *Volvox* show negative thermotaxis moving to an optimum of 5 °C to 10 °C,” based on publications from 1893 and 1928. It is possible that the tendency to migrate to lower temperatures may be a common feature with the order Chlamydomonadales. This preference for lower temperature contrasts with thermotaxis in *Paramecium*, which accumulate at their respective cultivation temperature^1^. *Chlamydomonas* cells did not migrate in large numbers toward 10 °C, as judged from the measurements of thermotaxis in the 10 °C–15 °C gradient. Previous work determined 15 °C to be the temperature at which *Chlamydomonas* cells can proliferate, although the proliferation rate is much slower than at 25 °C^31^. Soon after exposure to temperatures ranging from 4 °C to 9 °C, *Chlamydomonas* cell division pauses and cells accumulate starch and sugar for acclimation^14,32^. Thus, *Chlamydomonas* cells likely migrate toward low temperatures, as long as they can continue to proliferate but do not prefer the low temperature at which cell division halts.

Redox balance is altered by external factors and cellular activities, such as respiration and photosynthesis, and is detected by various signaling proteins, including ion channels and kinases^33^. The perturbation of redox balance could thus affect cell behavior. For example, *Chlamydomonas* cells display negative phototaxis under reducing conditions and positive phototaxis under oxidizing conditions^21^. Redox state also controls bacterial chemotaxis by alteration of CheA kinase activity^34^. In this study, we found that shifting the redox balance to reducing and oxidizing extremes suppressed thermotaxis in *Chlamydomonas*, except in cells grown at 20 °C. When the phototaxis result is compared with thermotaxis data obtained at comparable temperature (25 °C), the decline in thermotaxis in reducing and oxidizing conditions contrasts with augmentation of phototaxis at these extremes. *Chlamydomonas* cells appear to shut off thermotaxis and give priority to phototaxis in unusually reduced or oxidized states.

The possible causes for the observed biased distribution along the experimental temperature gradient include: (1) low motility at the site of cell accumulation; (2) change in the frequency of spontaneous backward swimming and/or swimming velocity; (3) steering of the swimming direction toward the preferred temperature. The second mechanism is involved in *Paramecium* and bacterial thermotaxis. The third mechanism is responsible for phototaxis in *Chlamydomonas*. We discuss these possibilities below.

On the whole, slower swimming at lower temperature is likely to contribute to negative thermotaxis because cells stay for a longer period where temperature is low^26^. Consistent with this idea, *C. moewusii* cells, which show positive thermotaxis, swim slowest at highest temperature examined^15^. However, thermotaxis is not fully attributable to swimming velocity. This is because the direction of thermotaxis reversed depending on redox state in cells grown at 20 °C without changing the sign of the slope in the relationship between temperature and swimming velocity. Additionally, cells grown at 15 °C exhibited a stronger thermotaxis response under ambient conditions than under oxidizing or reducing conditions, although the temperature dependency of swimming velocity did not significantly change. Lastly, the thermotactic profile predicted from swimming velocity did not reproduce the actual thermotactic profile.

*ptx1* cells showed thermotaxis, indicating that cell steering caused by Ca^2+^-dependent control of flagellar dominance is not involved in thermotaxis. The finding that thermotaxis does not require reorientation mechanism indicates that this behavior does not fall into “taxis” in a narrow sense, in which cells turn toward or away from stimulus as in phototaxis in *Chlamydomonas*^17^.

On the other hand, the absence of thermotaxis in *ppr2* and *ppr3* indicates that backward swimming or components used in backward swimming is involved in thermotaxis. The reversal of swimming direction in *Chlamydomonas* cells is induced by a Ca^2+^ influx through a voltage dependent Ca^2+^ channel in the flagellar membrane, but this flagellar current is absent in *ppr2* and *ppr3* mutants^27,28^. Interestingly, the frequency of spontaneous backward swimming increased with temperature in a condition where negative thermotaxis occurs but not under weak positive thermotaxis condition. Since pivoting motion at the end of backward swimming randomizes the direction of swimming, it is plausible that cells far from preferred temperature gain a higher chance to alter the swimming direction than the cells close to preferred temperature. The spontaneous backward swimming was, however, suppressed in *Chlamydomonas* cells when temperature was changed temporally. Thus, *Chlamydomonas* cells probably respond to static temperature rather than a temporal change in temperature whereas *Paramecium* and bacterial cells respond to a temporal change in temperature^3,4,35,36^. Suppression of spontaneous backward swimming upon temporal change in temperature suggests that swimming path would not be interrupted by spontaneous backward swimming when cells swim parallel to the temperature gradient but that they undergo spontaneous backward swimming more frequently when they swim vertically to the gradient.

It is of interest how *Chlamydomonas* cells detect temperature. The 0.14 °C/mm gradient used in this study is not subtle, considering that sperm undergo thermotaxis with a 0.014 °C/mm temperature gradient. Sperm thermotaxis involves TRP channels and opsins^6–9^. *Chlamydomonas* cells also express multiple TRP channel and opsin genes. At least eight TRP channels are expressed, including PKD2, TRP11, and ADF1, which are involved in mating, mechanoreception, and deflagellation, respectively^25,37,38^. Eight opsins are expressed, including the channelopsins, ChR1 and ChR2, which generate photoreceptor current at the eyespot^39,40^. However, the activity of TRP channels, opsins, and other proteins in thermosensing requires further study.

This study has shown that *Chlamydomonas* cells migrate predominantly toward the lower temperature in ambient condition. Thermotaxis is not a simple response to temperature, but is facilitated, inhibited, or even inverted by physiological conditions such as redox state and cultivation temperature. Our work sheds light on the dominance of redox balance on how cells control their motility and decide behavioral strategy.

## Methods

### Cell and culture condition

We used *Chlamydomonas reinhardtii* 137c mt+ (CC125) as wild type in our study. We also used the following mutant strains. *pf17* is a mutant with paralyzed flagella^41^. *ppr2* and *ppr3* are mutants that do not show photophobic responses^27^. *ptx1* is a mutant defective in phototaxis^19^. Cells were grown in Sager and Granick liquid culture medium^42^ under 12-hour light, 12-hour dark cycle at 15 °C, 20 °C, or 25 °C for 3 or 4 days.

We washed cells twice with a solution containing: 1 mM KCl, 0.3 mM CaCl_2_, 0.2 mM EGTA-KOH, and 5 mM HEPES-KOH (pH 7.4). The cell density was adjusted to 3 × 10^6^ cells/ml. Cells were kept at the cultivation temperature throughout the preparation process.

We included 0.2 mM *t*-BOOH or 75 mM DMTU in the experimental solution to assess thermotaxis under oxidizing or reducing conditions, respectively, according to Wakabayashi *et al*.^21^.

### Thermotaxis assay

We generated the temperature gradient on a 0.3-mm-thick brass plate that was placed between two Peltier devices (Supplementary Fig. 3). We monitored the temperature on the Peltier devices using platinum temperature sensors and controlled the temperature with a digital proportional integral controller (PLC-24V6A, T. S. Laboratory, Japan). We placed two complementary metal oxide semiconductor (CMOS) temperature sensors at positions that trisect the trough to check the temperature gradient along the brass plate. As shown in Supplementary Table 1, the temperatures at the CMOS sensors confirmed that a continuous temperature gradient was generated between Peltier devices. A water-soaked filter paper was placed between the brass plate and trough for better thermal conductivity, as well as providing a uniform matte background for observation. The bottom of the trough was made of a thin glass (0.12 to 0.17 mm thick) and the inside dimensions of the trough were 31 × 5 × 5 mm (length × width × height). The brass plate and trough were covered with Styrofoam for thermal insulation. The entire experimental set-up was placed in a dark box.

For the thermotaxis assay, we placed 1.1 ml of cell suspension in the trough at cell density of 3 × 10^6^ cells/mL. We illuminated the cells with red light (λ > 600 nm) because cell motility tends to decline in total darkness. Red light does not promote phototaxis^43^. We changed the direction of the temperature gradient between experiments to avoid the influence of factors independent of the temperature gradient.

We recorded the cell distribution with a charge-coupled device (CCD) camera and converted the image to gray scale (larger and smaller value indicate brighter and darker images, i. e. less and more cells, respectively). Gray scale value changed almost linearly with cell concentration in a range from 1.3 × 10^5^ to 1.2 × 10^7^ cells/mL (Supplementary Fig. 4). We divided the trough into five sections along its length and averaged the gray scale of each section. The value was normalized to 1 with the average gray scale at time = 0 min, to yield G_i_ (i = 1 to 5). The percent cell density in each section was expressed as

1 $$Ci=100\times \{1+(1-\frac{5Gi}{{\sum }_{j=1}^{5}\,Gj})\}$$

Thermotactic index (TI) was defined as

2 $$TI=\frac{{\sum }_{i=1}^{5}\,SiCi}{5}$$

where S_i_ is 2 (highest temperature), 1, 0, −1, and −2 (lowest temperature) in the order of sections with higher temperature. Thus, positive TI represents accumulation toward warmer sections (positive thermotaxis).

### Swimming velocity

We recorded swimming velocity at different temperatures using an inverted microscope (IX70, Olympus) equipped with a temperature-controlled stage (Thermo Plate, Tokai Hit, Japan). We acquired the video using a CCD camera (XC-EI50, Sony) connected to a personal computer (Apple Computer) through a video converter (ADVC-55, Canopus, Japan). We used red light (λ > 600 nm) for illumination to avoid a photoresponse^43^. Swimming velocity was calculated by dividing displacement of a cell by the time of measurement using ImageJ.

The frequency of spontaneous backward swimming was recorded in the same way except that the distance between the slide and cover glass was set at approximately 100 µm by adjusting the volume of cell suspension. We set the focus in the middle between the slide and cover glass so as not to count the avoiding reaction, which occurs when cells collided with the glass surface.

## Electronic supplementary material

Supplementary Information

## Data Availability

All data generated or analyzed during this study are included in this published article and the Supplementary Information.
