# Supplementary material for: Thermotaxis in Chlamydomonas is brought about by membrane excitation and controlled by redox conditions
Source: Sci Rep. 2018 Oct 31;8:16114. doi: 10.1038/s41598-018-34487-4 (PMC6208428; doi:10.1038/s41598-018-34487-4)
Supplement: Supplementary file 1 — Supplementary Information [file 41598_2018_34487_MOESM1_ESM.docx]

**Supplementary Information for**

**Thermotaxis in *Chlamydomonas* is brought about by membrane excitation and controlled by redox conditions**

Masaya Sekiguchi, Shigetoshi Kameda, Satoshi Kurosawa,

Megumi Yoshida, and Kenjiro Yoshimura*

Department of Machinery and Control Systems, College of Systems Engineering and Science

Shibaura Institute of Technology, Saitama 337-8570, Japan


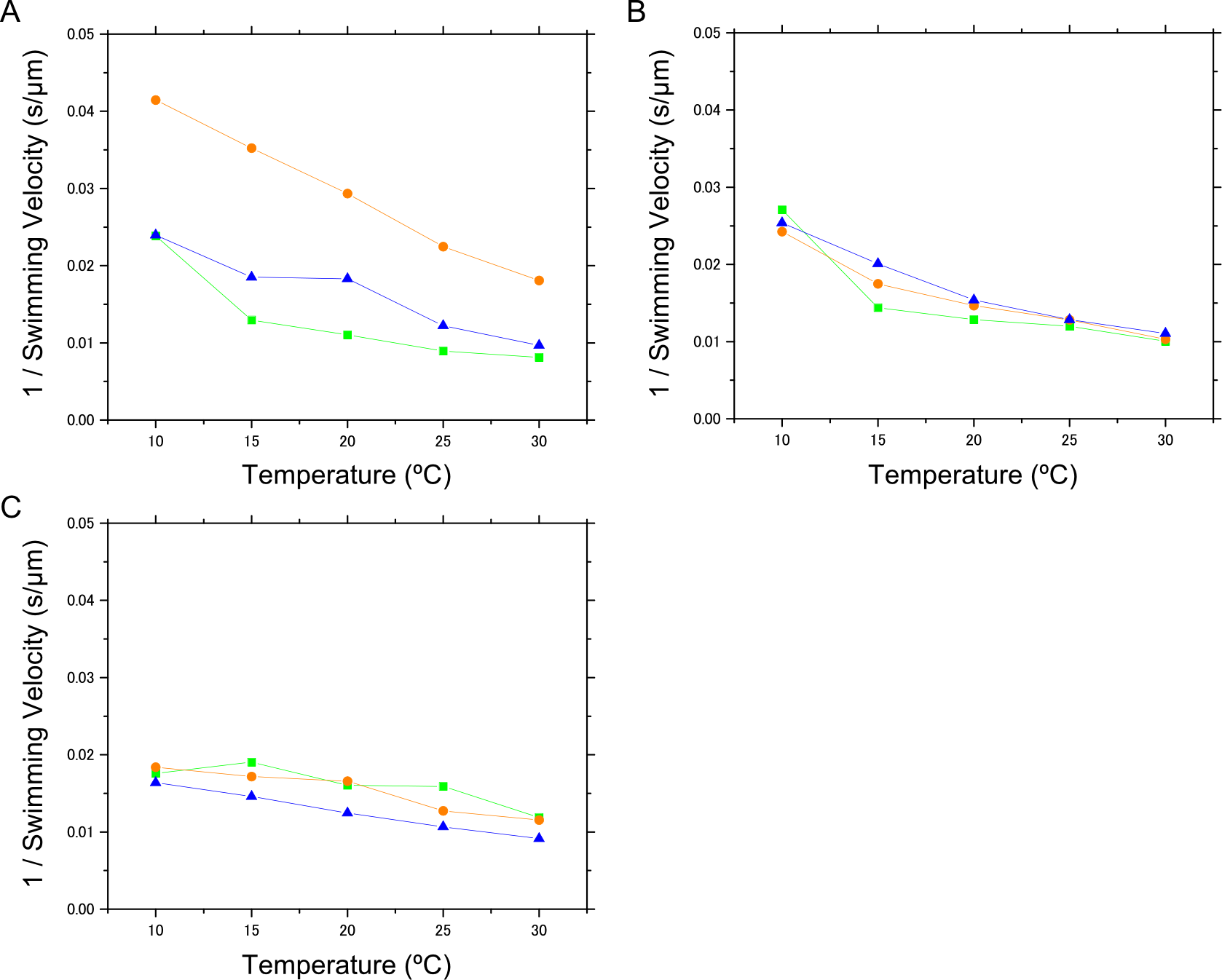


**Supplementary Figure 1** Correlation of the inverse of swimming speed with temperature. The data shown in Fig. 4 are used. Wild type cells were grown at (A) 25ºC, (B) 20ºC, and (C) 15ºC. Data obtained under ambient (green), reducing, (blue), and oxidizing (orange) conditions are shown.


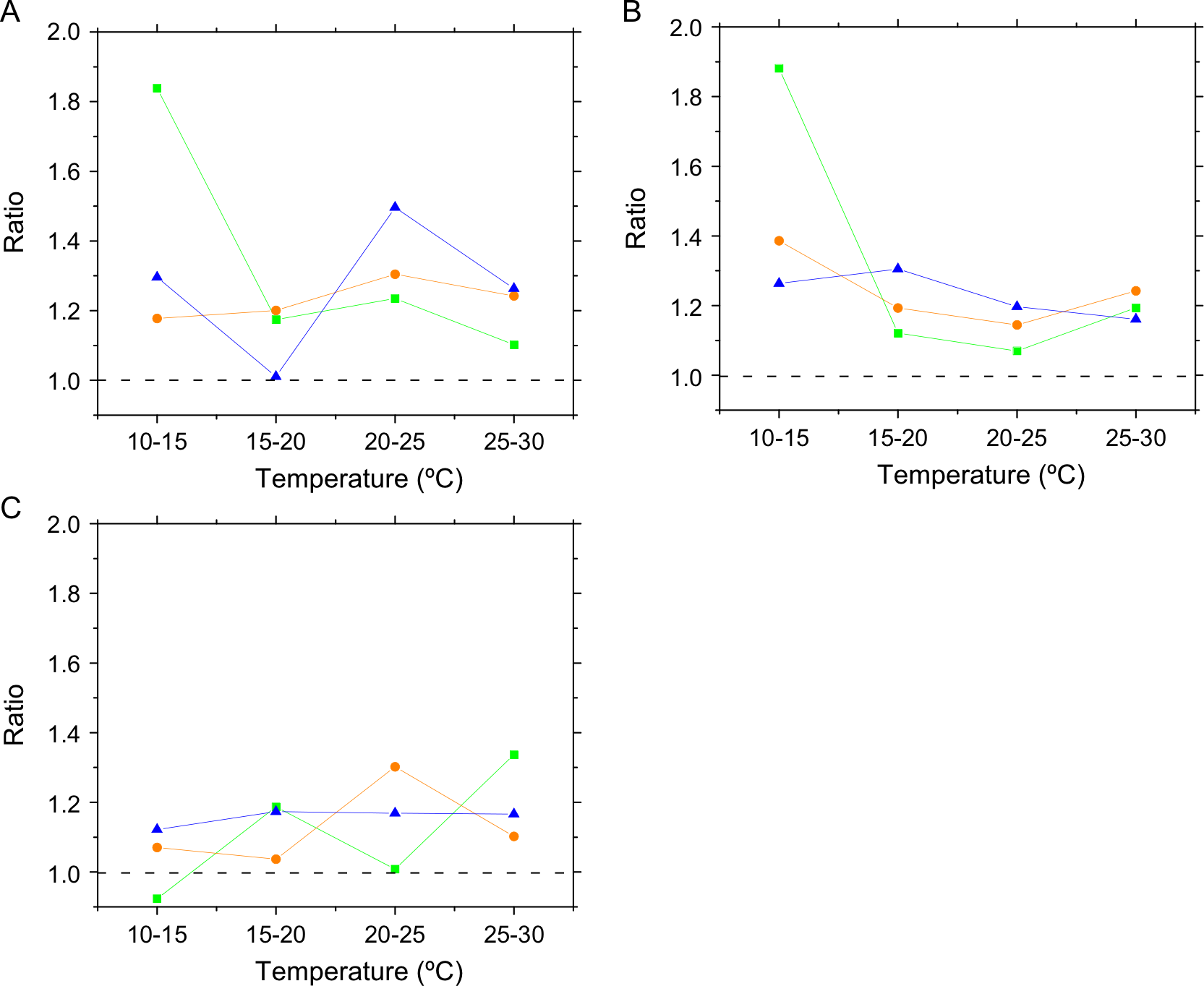


**Supplementary Figure 2** The ratio of the inverse of swimming speed at two different temperatures. The temperatures are shown on the horizontal axis. The data shown in Supplementary Fig. 1 are used. Wild type cells were grown at (A) 25ºC, (B) 20ºC, and (C) 15ºC. Data obtained under ambient (green), reducing, (blue), and oxidizing (orange) conditions are shown.


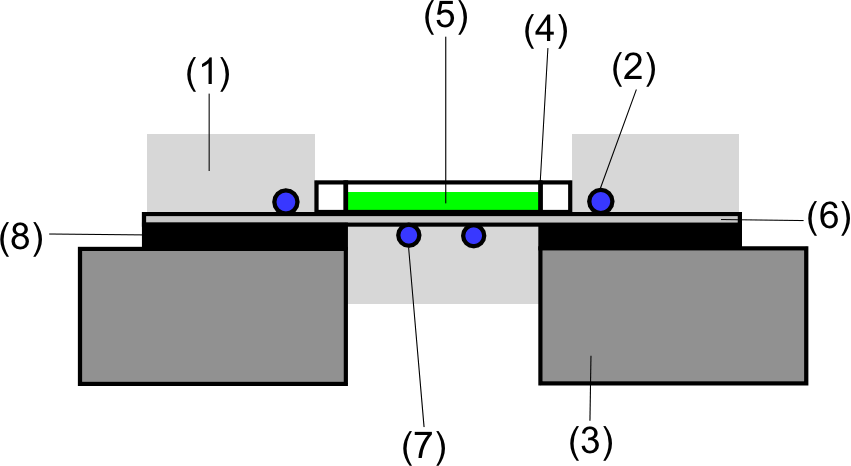


**Supplementary Figure 3** Experimental set-up to assess thermotaxis. (1) Styrofoam, (2) platinum temperature sensors, (3) heat sinks, (4) trough, (5) cell suspension, (6) a brass plate, (7) complementary metal oxide semiconductor (CMOS) temperature sensors, and (8) Peltier devices.


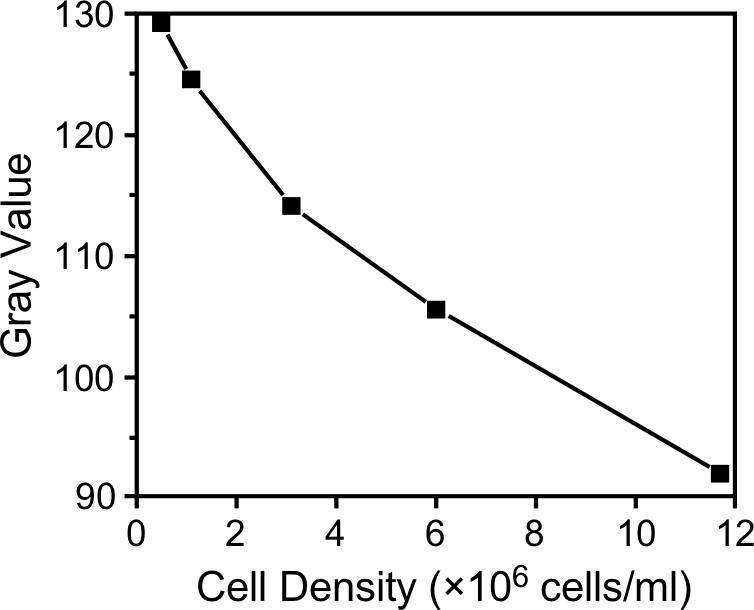


**Supplementary Figure 4** Correlation between cell density and gray scale value.

**Supplementary Table 1** Temperature (ºC) along trough

| Peltier device Sensor A Sensor B Peltier device  temperature temperature |
| --- |
| 10.0 12.5±0.9 13.9±0.9 15.0  15.0 17.5±0.7 19.0±0.6 20.0  20.0 21.9±0.5 23.6±0.5 25.0  25.0 26.3±0.6 28.0±0.6 30.0 |

Sensor A was closer to the Peltier device and set at the lower temperature, and Sensor B was further from the Peltier device and set at the higher temperature. Mean ± SD of 64 or 65 measurements.
